# The human coronaviruses (HCoVs) and the molecular mechanisms of SARS-CoV-2 infection

**DOI:** 10.1007/s00109-020-02012-8

**Published:** 2020-12-02

**Authors:** Luigi Santacroce, Ioannis A. Charitos, Domenico M. Carretta, Emanuele De Nitto, Roberto Lovero

**Affiliations:** 1 Department of Interdisciplinary Medicine, Microbiology and Virology Laboratory, University Hospital of Bari, Università degli Studi di Bari, p.zza G. Cesare, 11, 70124 Bari, Italy; 2 Department of Emergency and Urgency, National Poisoning Centre, Riuniti University Hospital of Foggia, viale Pinto, 1, Foggia, 71122 Italy; 3 Syncope Unit at Cardio-Thoracic Department, Policlinico Consorziale, U.O.S. Coronary Unit and Electrophysiology/Pacing Unit, p.zza G. Cesare 11, Bari, 70124 Italy; 4 Department of Basic Medical Sciences, Neuroscience and Sense Organs, Section of Biochemistry, University of Bari “Aldo Moro”, p.zza G. Cesare, 11, 70124 Bari, Italy; 5 Clinical Pathology Unit, AOU Policlinico Consorziale di Bari - Ospedale Giovanni XXIII, p.zza G. Cesare 11, 70124 Bari, Italy

**Keywords:** Coronavirus, SARS-CoV-2, Pandemics, Molecular biology, Immunity, Pathology, Human microbiota

## Abstract

In humans, coronaviruses can cause infections of the respiratory system, with damage of varying severity depending on the virus examined: ranging from mild-to-moderate upper respiratory tract diseases, such as the common cold, pneumonia, severe acute respiratory syndrome, kidney failure, and even death. Human coronaviruses known to date, common throughout the world, are seven. The most common—and least harmful—ones were discovered in the 1960s and cause a common cold. Others, more dangerous, identified in the early 2000s and cause more severe respiratory tract infections. Among these the SARS-CoV, isolated in 2003 and responsible for the severe acute respiratory syndrome (the so-called SARS), which appeared in China in November 2002, the coronavirus 2012 (2012-nCoV) cause of the Middle Eastern respiratory syndrome (MERS) from coronavirus, which exploded in June 2012 in Saudi Arabia, and actually SARS-CoV-2. On December 31, 2019, a new coronavirus strain was reported in Wuhan, China, identified as a new coronavirus beta strain ß-CoV from group 2B, with a genetic similarity of approximately 70% to SARS-CoV, the virus responsible of SARS. In the first half of February, the International Committee on Taxonomy of Viruses (ICTV), in charge of the designation and naming of the viruses (i.e., species, genus, family, etc.), thus definitively named the new coronavirus as SARS-CoV-2. This article highlights the main knowledge we have about the biomolecular and pathophysiologic mechanisms of SARS-CoV-2.

## Introduction

In recent years, coronaviruses have been responsible for the two pandemics: that of severe acute respiratory syndrome (SARS COVID-1), which began in 2002 and is currently at the end of 2019, following a new strain, the SARS-CoV-19 [1] The virus was first detected in the Wuhan region of China and caused a pandemic spread to over 114 countries around the world. Coronaviruses are a group of viruses that often cause mild respiratory infections in humans and animals. Most people become infected with coronavirus at least once in their lifetime, having mild-to-moderate symptoms of the common cold [2–4]. *Coronaviruses* show a notable propensity to transmit themselves to new hosts, and this propensity has emerged on several occasions in the evolutionary history of this viral family; suffice it to recall the human coronavirus HCoV-OC43, which has close genetic correlations with the *bovine coronavirus* (BCoV), and which seems to derive from this or the human coronavirus HCoV-229E which after originating from a bat *Alphacoronavirus* has passed back to an animal host generating the swine epidemic diarrhea virus (PEDV), or the most important human coronavirus, SARS-CoV and SARS-CoV-2, which seems to have its reservoir in bats. In fact, the sequences of the Wuhan beta coronavirus shows similarities to the *Betacoronaviruses* found in bats. Thus, bats are the natural reservoir of numerous *Alphacoronaviruses* and *Betacoronaviruses* [5–10] (Table 1).

**Table 1:** The seven pathogenic human coronaviruses identified (HCoVs)

Strain	Genus/subgenus	Infect
MERS-CoV (Middle East respiratory syndrome coronavirus infection)	Betacoronavirus/Merbecovirus	Humans, bats, camels
SARS-CoV (severe acute respiratory syndrome coronavirus infection)	Betacoronavirus/Sarbecovirus	Humans and mammals
Human coronavirus 2019 HCoV-19 or SARS-CoV-2 (severe acute respiratory syndrome coronavirus 2)	Betacoronavirus/Sarbecovirus	Humans
HCoV-HKU1 (human coronavirus HKU1)	Betacoronavirus/Embecovirus	Humans, mice
HCoV-229E (human coronavirus 229E)	Alphacoronavirus/Duvinacovirus	Humans, bats
HCoV-NL63 (human coronavirus NL63)	Alphacoronavirus/Setracovirus	Humans
HCoV-OC43 (human coronavirus OC43)	Betacoronavirus/Embecovirus	Humans, cattle

In fact, these viruses were previously called SARS-like CoV. The SARS-like CoVs, along with human and animal SARS-CoVs, have been included in the genus *Betacoronavirus*, in a taxonomic grouping created especially for them, the severe acute respiratory syndrome-related coronavirus (SARS-related CoV). SARS-like CoVs in the past have been identified in Asia, Europe, and Africa. The finding of various strains of SARS-like CoV in the beards of several *Rhinolophus affinis*–related species suggested that this group of coronaviruses is rapidly evolving and can overcome the species barrier. However, some strains of SARS-like CoV have been identified in hosts other than *Rhinolophus* spp. [11, 12]

## General virus features

### a) The viral evolution and *coevolution*

Evolution is the genomic modifications that a population acquires over time; in the case of viruses, they do not only depend on the characteristics of the viral population but also on the physical-biological characteristics of the cell and the structure of the host population. The main success factor of viruses is, despite the small number of genes possessed, the high genetic variability that provides them with the ability to adapt and survive (Table 2) [13].

**Table 2:** Main definition for viruses

Properties of viruses	
They are not living beings	
To exist in nature, they must be infectious	
They must use host cell mechanisms to produce their own components (viral mRNA, proteins, and identical genome copies)	
They must encode their own specific proteins for each process they request and not carried out by the cell	
The production of new virions occurs by assembly of viral components	

In general, viral evolution can follow two paths: (a) viral population coevolves with the host following a single fate; if the host thrives, the virus thrives too. However, since there are no host alternatives for the virus, a factor may arise that prevents replication of the virus in that host (e.g., antiviral), and therefore, the viral population dies out (more common for DNA viruses); (b) viral population expands into multiple biological niches by infecting different hosts. If a factor arises that prevents the virus from replicating in one host, the virus replicates in another and the population does not become extinct (more common for RNA viruses such as the *Betacoronavirus* and others) [1].

Selection is the process by which a genotype is fixed or not in the population through the evaluation made by the environment of the phenotype it encoded. For evolutionary purposes, the main characteristics of the virus-receptor interaction are: (a) a virus can use different receptors and co-receptors; (b) the same receptor can be used by different viruses and other microorganisms; (c) the type of receptor used by a virus cannot be predicted on the basis of its phylogenetic position or biological properties; and (d) one or a few mutations of the viral genome can modify the receptor used, the cell tropism, and the pathogenicity [13–15].

By coevolution, we mean a process in a dynamic equilibrium (which varies over time) of reciprocal adaptation, by means of genetic modification, between two or more species. In this process, the genetic modifications of one species are the result of the selection made by the other species and vice versa. In the co-evolutionary process, a pathogen and its host are closely related and mutually adapt in opposite directions, just think of some characteristics such as the infectivity of the pathogen and the resistance of the host, or the response of the host to infection and immune evasion by the pathogen. In this context, virulence, e.g., the damage manifested by the host in response to the infection given by the pathogen, is an ambiguous manifestation of the state of coevolution between the two, since it manifests a state of poor fitness of the host that is not always accompanied by an efficient completion of the pathogen replication cycle; it often happens that the adaptation of a virus in a host leads to an attenuation of its virulence [16, 17].

### b) The genetic variation of viruses

The genetic variation is the process, devoid of specific and predetermined purposes, which underlies the differentiation and therefore the evolution of viruses. The set of viral variants that originate because of different molecular mechanisms allow the virus to adapt to the environment it will act by selecting the form, or viral type, with the winning characteristics. Viruses use the same molecular mechanisms of genetic variation as other life forms: mutation, recombination, and gene reassortment [2].

Mutation is a copy error of the nucleic acid by RNA or DNA polymerase, action of enzymes of the host cell; chemical-physical damage of the nucleic acid and can be divided into three main types: (a) transitions: replacement of a purine with a purine or of a pyrimidine with a pyrimidine; (b) transversions: replacement of a purine with a pyrimidine or vice versa; (c) insertions or deletions (indel): addition or subtraction of one or more nucleotides. The mutation, understood as an event that occurs on a population (set of organisms or genes or traits of them), can be quantitatively expressed through two parameters, the mutation rate, and the mutation frequency. The mutation rate quantifies the number of mutations per single copied nucleotide, regardless of the fate of the error produced, so it is a measure of a biochemical event. The mutation rate of viruses is influenced by (a) whether the viral DNA polymerase has proofreading activity; (b) whether the virus codes for restorative proteins; (c) replicative mechanism of the virus; (d) intracellular site of virus replication; (e) genome length (on average, the mutation rate in the genome of RNA viruses is 10^−3^–10^−5^ nt/site, while that of DNA viruses is at least 300 times lower, 10^−8^–10^−9^ nt/site). [13, 15, 18] The quasispecies defined as the viral population within a host composed of a distribution of different but closely related mutants, subject to continuous processes of genetic variation, competition, and selection, which act as a selection unit. The quasispecies theory is suitable for explaining the cloud of viral variants that created in the host infected with viruses characterized by high mutation rates; RNA viruses (such as the coronaviruses) are therefore the main viruses to follow this dynamic, together with some DNA viruses with small genomes [19].

Recombination is a widespread mechanism of genetic variation that occurs for both RNA and DNA viruses and often with the participation of the replicative complex of the virus [12]. To have recombination, the same host cell must be coinfected by at least two different viral genomes; the persistence of a viral genome in the cell (persistent infection) favors the establishment of a co-infection for the subsequent penetration of another virus and therefore increases the probability of a recombination event. A recombination event is suspected when the phylogenetic analysis of the same viral strain shows discordant positions for different genes; in these cases, confirmation must be done by statistical evaluation to differentiate a true recombination from possible convergent mutations which arose independently. [14]

The gene reassortment is the exchange of genome fragments between two or more viruses (RNA or DNA) with a segmented genome that infect the same host cell. It is an evolutionary phenomenon that allows major changes and is greatly exploited by these viruses, just think of the frequent generation of new variants that occurs in this way for the flu virus. These different molecular mechanisms of genetic variation make it possible to distinguish two types of viral evolution which can be differentiated precisely by the molecular mechanisms involved, the times with which they occur and the effects they have on the virus. The “genetic drift,” is the progressive and scaling modification over time, by small successive steps, of the viral genome; it is the genetic diversity resulting from single mutations (due to duplication errors) that occur each time the genome replicates and from the subsequent selection made by the environment. The “genetic shift” is the massive and single-stage modification of the viral genome as a consequence of recombination or reassortment; it is the genetic diversity resulting from a relatively rare event that occurs only in certain circumstances [13].

### c) The cross-species transmission

When we refer to emerging viruses, we mean the causative agent of a new or never previously encountered viral infection in the population such as SARS-CoV-2. The emergence of the virus leads to an increase in the incidence of the disease caused by it in a new host, or in the traditional host following events that alter the epidemiology; in the latter case, it would be more correct to speak of re-emergence. Viruses undergo a process of random evolution resulting from the biological interactions they establish with the host, and, in conjunction with certain events or factors, this evolution can lead to the emergence of a disease. The emergence of new viral diseases can therefore affect the passage of a pathogen from one animal to another, or the passage of a pathogen from an animal to man. The emergence of new zoonoses represents the main threat to public health and are events that have repeated themselves with some frequency in the last decades, suffice it to recall the past SARS virus, the influenza, Nipah, Hendra, Ebola, and today the pandemic SARS-CoV-2 viruses [20–22].

**Fig. 1:**
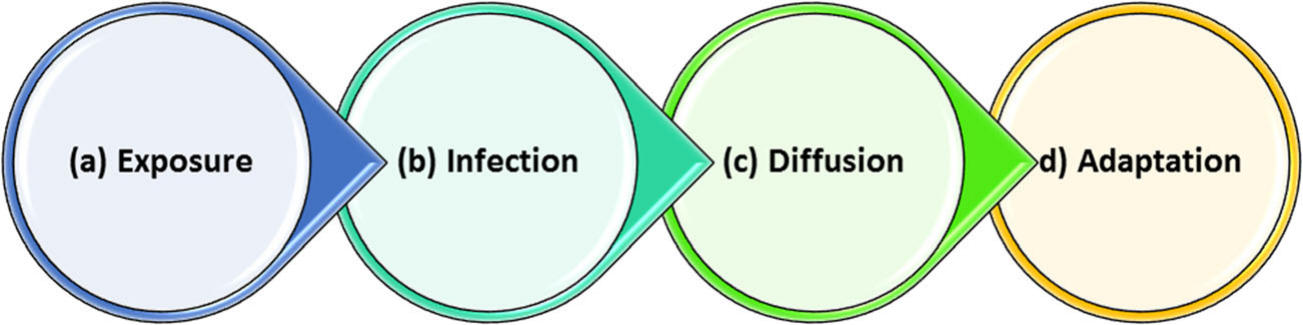
The emergence of a non-pre-adapted virus in a new host follows four consecutive phases: **a** Exposure (contact between the donor host and the recipient, which can be influenced by geographical, ecological, and behavioral factors). **b** Infection (the passage of the virus from the donor host to the recipient, which can be influenced by the ability of the virus to overcome the species barriers and by the compatibility with the new host: binding to the cell receptor, ability to complete the replication cycle, evasion the immune response, etc.). **c** Diffusion (transmission of the virus between subjects belonging to the new population, which can be influenced by the ability of the virus to complete its replication cycle in the new host and by the contact between the subjects that make up the new population). **d** Adaptation (evolution of the virus in the new host so as to remain in equilibrium within the population, which can be influenced by the genetic variability of the virus) [24]

To have a pandemic emergence of a disease (such as COVID-19), two elements are required: (a) the introduction of the pathogen into the population and (b) its consequent spread and maintenance in the population itself. The factors that can lead to the emergence of infectious diseases are different and genomic adaptability plays an important role [23]. For this reason, the more a virus can mutate and adapt, the greater its ability to pass on to a new host and establish a productive infection there. The other factors are mostly environmental, ecological, and sociological influences that affect the likelihood of potential pathogens to meet with new hosts; in fact, many of these factors alter the spread of pathogens and their hosts or vectors. The emergence of a non-pre-adapted virus in a new host follows four phases (Fig. 1) [14, 21, 24].

In general, the interactions between virus and host can therefore be divided into four types: **a** stable, keeps the virus in the environment (the host and viral populations are in equilibrium and are maintained in the environment), characteristic behavior of the virus in the primary host or reservoir; **b** evolving, passage of the virus from the traditional population to a new one, belonging to the same host species or to different species, with progressive adaptation of the pathogen; **c** dead-end or dead-end, passage of the virus from the traditional host to a new one in which the virus is unable to transmit effectively and therefore does not adapt, it may also be due to the high lethality of the virus which causing death too quickly the new host does not allow the transmission; **d** resistant: the infected host completely blocks the replication cycle of the virus (non-receptive host). To infect a new host, a virus must be able to overcome the species barriers and carry out its replication cycle in its cells; this process can be limited at various levels, including receptor binding, cell entry, replication, gene expression, and the spread of an infecting progeny [24, 25].

The various factors that influence the ability of the virus to transmit from one host species to another, such as the ability to bind to the cell receptor of the new host, are generally influenced by the phylogenetic constraints existing between the host species in question; in the sense that the more the original host and the new host are phylogenetically related, the higher the probability that host barriers will be successfully overcome (it is easier for a pathogen to be transmitted from one mammal to another mammal than from a plant or insect to a mammal) [26, 27]. The main factor influencing the contact between donor and recipient host is man, as population dynamics, commercial and non-commercial transport, action on the environment, and social behaviors modify the dynamics and interactions, among others. Some animal species create potentially dangerous promiscuity situations. Once the virus has acquired the ability to infect the new host, further evolutionary adaptations may be necessary to have the pathogen spread; in fact, there are cases of pathogens that replicate and give disease in a new host but are not able to transmit from this to other subjects of the same or different species [14, 23].

Ultimately, the probability that the adaptation of the virus to the new host takes place successfully and that the pathogen therefore remains in it depends on four factors [22]: (a) the number of primary infections, determined by the amount of virus that is transmitted to the new host, by the number of infected individuals and by the repetition of multiple transmissions between the primary and new host; (b) the higher the value of R0 in the new host at the beginning of the transmission, the greater the probability; (c) the number of mutations or genetic changes necessary for the virus to adapt to complete its replicative cycle in the new host; (d) the specific characteristics of the pathogen, i.e., the genetic variability of the virus, the probability that the necessary mutations will occur, and how much R0 will change with each mutation [27, 28].

## Virological concepts of SARS-CoV-2

### a) Viral features

The RNA viruses, such as the coronaviruses, show a much higher evolutionary speed than DNA viruses, because of the high susceptibility to replication error mediated by RNA polymerase or reverse transcriptase, by the considerable size of the viral population and by the higher replication speed. These rapid evolutionary modifications allow RNA viruses to quickly generate mutations that can allow them to adapt to a new environment, including a new host species. If RNA viruses show an extreme variability, even the most complex DNA viruses still show a certain ability to generate different variants; the latter do not exhibit high mutation rates like RNA viruses since this would affect their functionality, but are subject to variation of specific sites called “hot spots.” Viruses with genetic material composed of a single small DNA strand which, probably, due to a high replication error rate, can reach an evolutionary speed equal to that of RNA viruses are an exception. The quasispecies theory is suitable for explaining the cloud of viral variants that created in the host infected with viruses characterized by high mutation rates; RNA viruses are therefore the main viruses to follow this dynamic, together with some DNA viruses with small genomes [19].The evolution of coronaviruses and therefore of COVID-19 occurs not only by nucleotide mutations but also by recombination. Although some nucleotide mutations are widely spread in the population, no univocal conclusions have been drawn regarding whether these mutations are responsible for the difference in the virulence of SARS-CoV-2. The number of replacements in the SARS-CoV-2 genome is about 26/year and depending on the size of the genome (29.9 kb), and the virus has an evolutionary rate of about 0.90 × 10^3^ replacements/site/year [29]. In an RNA virus with a genome of ten thousand bases, there will therefore be an average of one mutation for each replicated genome; therefore, in millions of progeny, there will be millions of mutations that create a population of mutants, all very similar but still different. In particular, SARS-CoV-2 such as an RNA virus replicates with a level of fidelity close to the error threshold, and in many cases, even if these viruses are potentially very variable, the small size of their genome limits the number of modifications and the positions of the genome in which they can occur without causing functional loss [13, 14, 17, 29].

In the case of viruses, emergence with pandemic effects is more frequently associated with RNA viruses, such as the *Betacoronaviruses*, that manifest more than other viruses, probably thanks to their greater genomic flexibility, mutation, recombination, and reassortment phenomena that allow them to overcome species barriers, adapt to different hosts, and give rise to new variants which will have isolated events. On the other hand, the primary host will always be needed to have the infection of the new one, while, in the second case, the pathogen will stabilize in the new population without needing the primary host and an event will develop epidemic that may become endemic [24]. The main obstacle to the emergence of a virus, following a jump in species, is therefore represented by the ability to obtain an efficient transmission of the virus itself between different individuals, from the infected to the healthy, belonging to the new host species. As we reported, a tool for assessing the possibility of emergence of a pathogen is the basic reproductive number (R0), which refers to the number of newly infected generated by an already infected individual in a completely sensitive population [24, 26]. This parameter depends on the number of infected-healthy contacts, the probability of transmission, and the contagiousness of the pathogen. R0 is a function of the balance that is created between the pathogen and the host and, therefore, represents a measure of the degree of adaptation that the pathogen shows in giving an effective infection in each host. For each pathogen, in a specific host of infection and at a precise moment, its R0 can be calculated, which will have high values depending on the adaptation of the virus in it (if R0 shows values of 1, the disease will spread in the population and the pathogen will stabilize in it; furthermore, if the R0 value is close to 1, the infection will have an endemic character, while if the value is greater than 1, the infection will have an epidemic character). For SARS-COVID-19, R0 reveals that for each infection directly generates 2–4 infections. But days of infectivity latency are variable from 3 or 4, and if use *R* = 4, the number of cases will quadruple [24, 26–28].

### b) The 2019-nCoV structure

The *Coronavirus* (CoV), order *Nidovirales* and family *Coronaviridae*, are a group of viruses equipped with single-stranded, linear not segmented genome, positive sense RNA (ssRNA +) and provided with envelope, which have a helical symmetry core and a characteristic crown morphology. Until a few years ago, all the viruses belonging to the family *Coronaviridae* were subdivided in only two genera: *Coronavirus* and *Torovirus*, and all the viruses classified in the genus *Coronavirus* were subdivided in their turn in only three antigenic groups (group I, group II, and group III). The recent discovery of a wide variety of new coronaviruses in different host species has prompted the Coronavirus Study Group of the International Committee for Taxonomy of Viruses (ICTV) to propose the reclassification of the *Coronaviridae* family into two subfamilies: *Coronavirinae* and *Torovirinae*. In particular, the subfamily *Coronavirinae* now includes three genera: *Alphacoronavirus*, *Betacoronavirus*, and *Gammacoronavirus* [14, 28, 30]. Although we still do not know the direct origin of SARS-CoV-2, most of the CoV belonging to the *Sarbecovirus* subgenus has been found in bats (such as horseshoe or other species). Therefore, it is possible that these bats could be the origin of SARS-CoV-2 [29]. SARS-CoV-2 has 29 proteins that can be of three types: structural, non-structural, and accessory (Table 3).

**Table 3:** The three types of SARS-CoV-2 proteins

SARS-CoV-2 proteins		
Structural	Non structural	Accessory
Glycoprotein S or Spike (S or ORF2), nucleocapsid (N or ORF9a), membrane (M or ORF5), envelope (E or ORF4)	NSP1, NSP2, NSP3, NSP4, NSP5, NSP6, NSP7, NSP8, NSP9, NSP10, NSP11, NSP12, NSP13, NSP14, NSP15, NSP16	ORF3a, ORF3b, ORF6, ORF7a, ORF7b, ORF8, ORF9b, ORF9c, ORF10

**Fig. 2:**
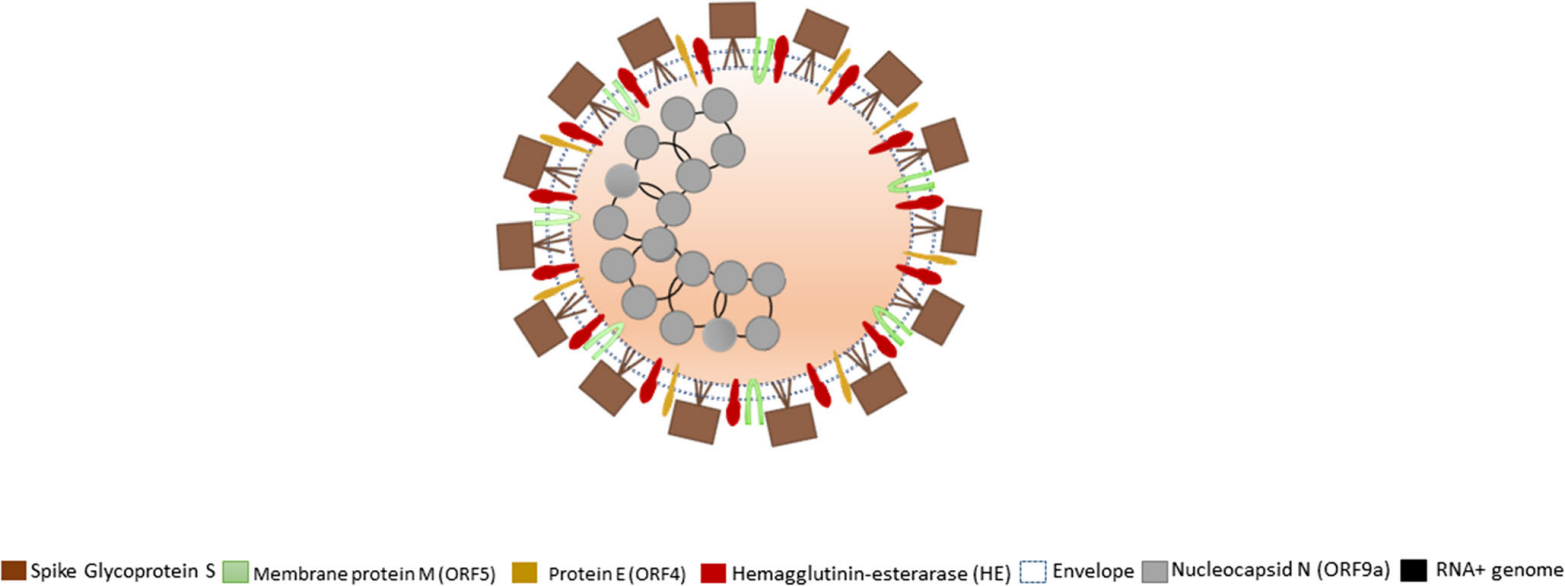
Coronavirus SARS-CoV-2 structure: glycoprotein S (spike) is cleaved into two glycosylated subunits, S1 (binds to the host’s receptor, ACE2) and S2 (aid viral and host membrane fusion). Membrane protein M aids to the assembly and budding of viral particles to ER-Golgi-intermediate compartment and interacts with ORF9a for RNA packaging into virion. Protein membrane E type III (single pass and forms a homopentameric ion channel, and is a viroporin) interacts with ORF5 and ORF9a, which aids in viral assembly, budding, and pathogenesis. Dimer hemagglutinin-esterase (HE) plays an important role during the release phase of the virus into the host cell. Genome consists of a single strand of large size positive sense RNA (26 to 32 kb in different viruses). Nucleocapsid N (ORF9a) plays its role in genome protection, viral RNA replication, virion assembly, and immune evasion (including IFN-I suppression). It binds to viral genomic RNA, forming a helical ribonucleocapsid and interacts with M and NSP3 proteins. Envelope is the coating of the virus, consisting of a membrane that the virus “inherits” from the host cell after infecting it

SARS-CoV-2 shows projections on its surface (about 20 nm long) formed by the glycoprotein S (spike) that join in groups of three that thus make up a trimer that together with the others form the structures that resemble a crown that surrounds the virion. The main differences of this new Coronavirus compared to SARS virus appear to be localized precisely in this spike protein and that glycoprotein S is the one that determines the specificity of the virus for epithelial cells of the respiratory tract, that is, to be able to bind the ACE2 receptor (angiotensin converting enzyme 2), expressed by the cells of the capillaries of the lungs, and studies have shown that SARS-CoV-2 has a higher affinity for ACE2 than SARS-CoV. Instead, the membrane protein M crosses the lining (envelope) interacting within the virion with the RNA protein and the protein E helps glycoprotein S to attach to the membrane of the target cell. The dimer-hemagglutinin esterase (HE) is a coating protein, smaller than glycoprotein S, and plays an important role during the release phase of the virus replication within the host cell. The envelope, which is the coating of the virus, consists of a membrane that the virus “inherits” from the host cell after infecting it. Coronaviruses are enveloped viruses that carry a genome of positive (+) sense RNA. RNA consists of a single strand of positive RNA of large size (27 to 32 kb in different viruses) and are not known larger RNA viruses. RNA originates seven viral proteins and is associated with protein N (nucleoprotein), which increases their stability (Fig. 2) [30–32].

The HCoV infectious cycle is initiated by the binding of virions to cell receptors, which will lead to a conformational change in the S2 subunit in S, thus leading to the condition of the fusion of the viral and cellular plasma membrane. In SARS-CoV-2, protein S cleavage and activation are regulated by transmembrane protease serine 2 (TMPRSS2) to generate fusion-catalyzed and unlocked forms on the cell surface. The gRNA will be the model for the translation of two polyproteins (pp1a and pp1ab), which in turn are broken down to form NSPS. Subsequently, the NSPS induces the reattachment of the cell membrane to form a double-membrane vesicle, where the viral RTCs (replication-transcription complexes) are anchored. The gRNA (full length) is replicated via a-gRNA intermediate, and a nested set of sgRNA species is synthesized by discontinuous transcription and that will encode the structural and accessory viral proteins. Subsequently, the viral products will be assembled in the ER/Golgi intermediate compartment where a smooth-walled vesicle will be created and will be directed towards the plasma membrane to exit via exocytosis and so on; again, another cycle will be created [33]. The measure of nucleotide-level genomic similarity between the coding regions of two genomes (nucleotide identity) of SARS-CoV-2 is 96% Bat CoV RaTG13, 93% Bat CoV RmYN02, 90% Pangolin CoV, 80% SARS-CoV, and 50% MERS-CoV belonging to Merbecovirus [29]. In a large analytical study of 10,022 SARS-CoV-2 genomes collected from 68 countries (mostly from the USA, the UK, Northern Ireland, and Australia), they detected a total of 65,776 variants, showing 5775 distinct variants (Table 4) [34].

**Table 4:**
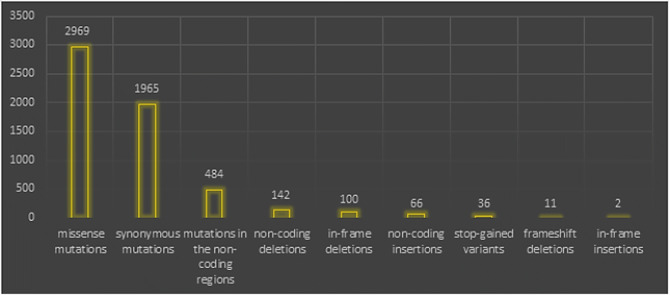
The 5775 distinct type variants out of 10,022 SARS-CoV-2 genome studies

**Fig. 3:**
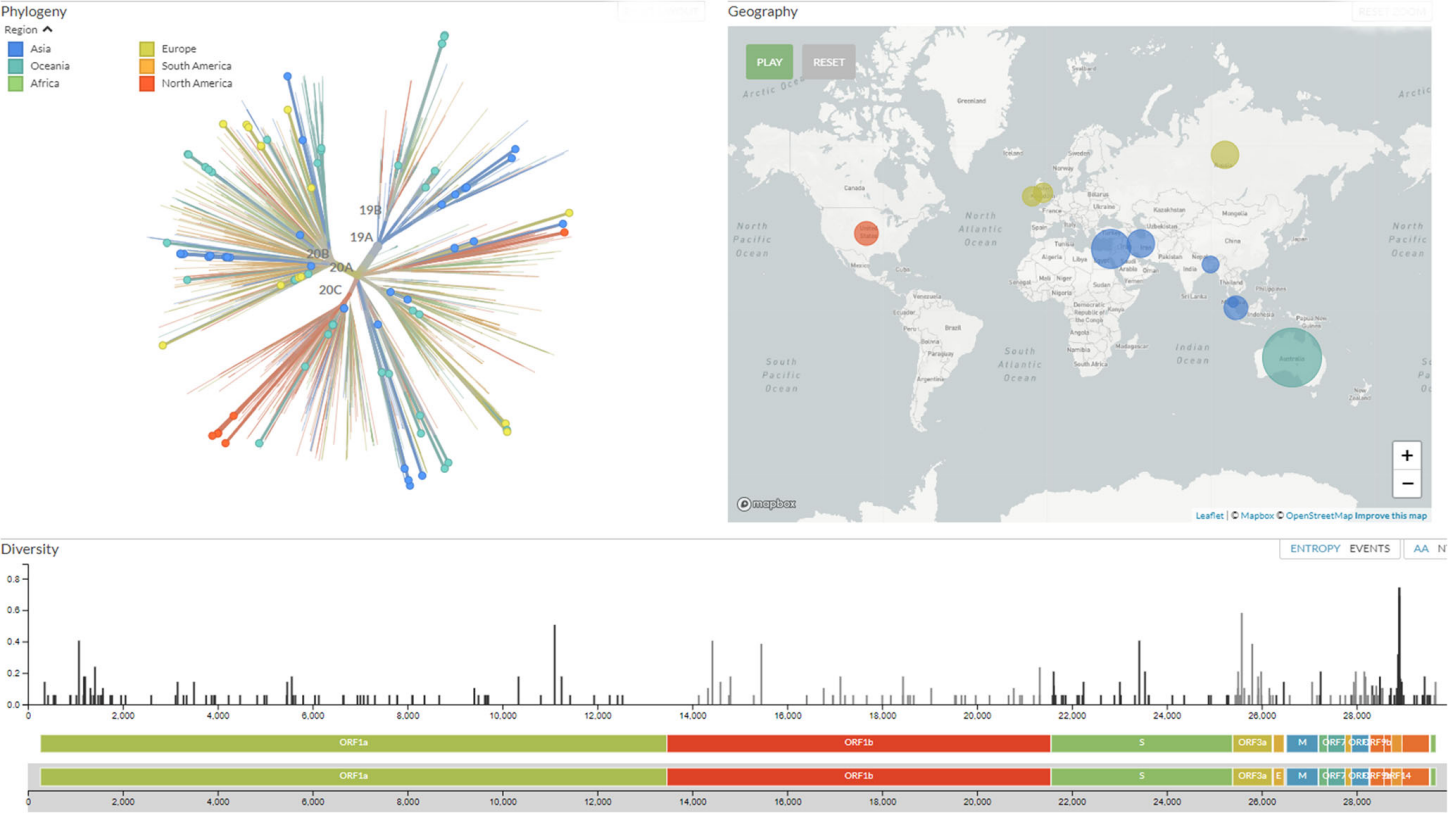
The genomic epidemiology of novel coronavirus (Global subsampling, 93 of 3587 genomes sampled between Mar 2020 and Oct 2020, source https://nextstrain.org/ncov/global?f_recency=1-2%20days%20ago&l=unrooted)

A new variant of the spike protein D614G that increases infectivity and transmissibility has been immersed. Thus, D614G is associated with potentially high viral loads in the patients but not with disease severity. The mutation that modifies the amino acid D614G is passed on as part of a conserved haplotype defined by four mutations that almost always follow together [35, 36]. Recently, an analysis of the number of variants in the open reading in frame 1ab of SARS-CoV-2 genomes, by finally cleaved protein of who showing deletions in non-structural proteins NSP1, NSP4, NSP6, NSP8, RdRp, NSP14 (exon N), endoRNase, and OMT was reported. One of the most important determinants of the pathogenicity of SARS-CoV-2 is the NSP1 protein, and this is quite new, as coronaviruses undergo a moderate rate of mutations, due to NSP4 with proofreading activity. It promotes viral gene expression and immune evasion in part by interfering with interferon-mediated signaling. It probably blocks host translation by interacting with the 40S ribosomal subunit, thus leading to degradation of host mRNA. The data clearly identify the new SARS-CoV-2 viral strain present in individuals from different areas of the world such as Europe and North and South America (Fig. 3) [32–39].

### c) Bio-pathogenesis and the immune responses

*Coronaviruses* infect numerous animal and human hosts and are characterized by a remarkable frequency of recombination, which, together with the high rate of mutation, encourage their adaptation to new hosts and ecological niches [13]. In most patients, the SARS-CoV-2 virus is attacked by the immune system and patients exhibit a wide range of variability in disease severity ranging from asymptomatic to extremely critical. The transmission occurs mainly through secretions and fecal excretions. The upper and lower airways are early involved in SARS-CoV-2 infection, then other systems such as the gastrointestinal, cardiovascular, renal, integumentary systems, with variable clinical manifestations; the central nervous system (CNS) and peripheral nervous system (PNS) can also be involved through synapses, so psychiatric/mental disorders can arise [40–42]. Respiratory infective Flügge drops infect the cells of epithelia/endothelia in the upper respiratory tract and progresses to lower regions of the lung and essentially causing respiratory damage of varying severity, as well as pulmonary macrophages (containing angiotensin-converting enzyme 2), neurons, microglia, and other. The life cycle of SARS-CoV-2 when it infects a person follows five steps: attachment, penetration, biosynthesis, maturation, and release. Once the viruses bind to the receptors, they enter the host’s cells [39, 42–47]. As we mentioned, protein modeling experiments on virus protein S have suggested that SARS-CoV-2 has affinity with enzyme 2 receptors of human cell angiotensin conversion enzyme 2 (ACE2) to use it as an entry “port” into the cell, and each SARS-CoV-2 virion is approximately 50–200 nm in diameter [31, 40].

The virus escapes the control of the immune system and decreases the number of lymphocytes so that they do not eliminate the virus, and monocytes/macrophages are not regulated and start pumping “cytokine storms” (neutrophil-to-lymphocyte ratio (NLR) is the leading indicator of hypercytokinemia). The virus’ direct pro-inflammatory effect response can cause acute damage to lung tissue and, subsequently, the hyper-inflammation leads to the acute respiratory distress syndrome. The development of SARS-CoV-2 infection towards the lower airways depends on the damage caused to the bronchioles which disrupts the protective coatings of the surfactant released by type II pneumocytes. This leads to alveolar alteration and desquamation of the endothelial cells and therefore impaired gas exchange and subsequent hypoxemia which will lead to acute respiratory distress syndrome (ARDS). But ARDS itself will in turn cause an overactive immune defect (by localizing neutrophils and increasing cytokines), which will lead to an increase in free radicals, cell debris and proteases, and edema resulting from the increase in proteins in the interstitial space, and the consequent vasoconstriction through platelet activation that further alters alveolar gas exchange with severe hypoxemia and tissue hypoxia which will lead to multi organ failure (MOF) [31, 43, 48]. The mechanism of thrombocytopenia in patients then has a multifactorial origin. Furthermore, the combination of viral infection and mechanical ventilation can give additional endothelial damage which will also lead to platelet thrombosis in the lung, thus causing excessive consumption of platelets, and a decrease or capillary morphological alternation can lead to altered platelet defragmentation. But another factor would be that coronaviruses can infect the bone marrow resulting in the activation of an autoimmune response against blood cells [42, 46, 49–51]. Histological examination of the lungs of patients who died from ARDS shows diffuse alveolar damage with desquamated pneumocytes II and lymphocytic infiltrates (CD4 and CD8 lymphocytes) mainly located in the interstitial spaces, around the larger bronchioles, and in many cases, there are foci of hemorrhage, and small vessels appearing to contain platelets and small thrombi. The CD4 and CD8 cells (killer lymphocytes) collaborate with B lymphocytes responsible for the production of antibodies. The CD8 destroys the infected cells by cytolysis and necrotizing cytokines instead of the CD4, and releases interferons and interleukins which eliminate the pathogens (beneficial effect) but, on the other hand, may also have harmful effects (immunopathology) [52–54].

**Fig. 4:**
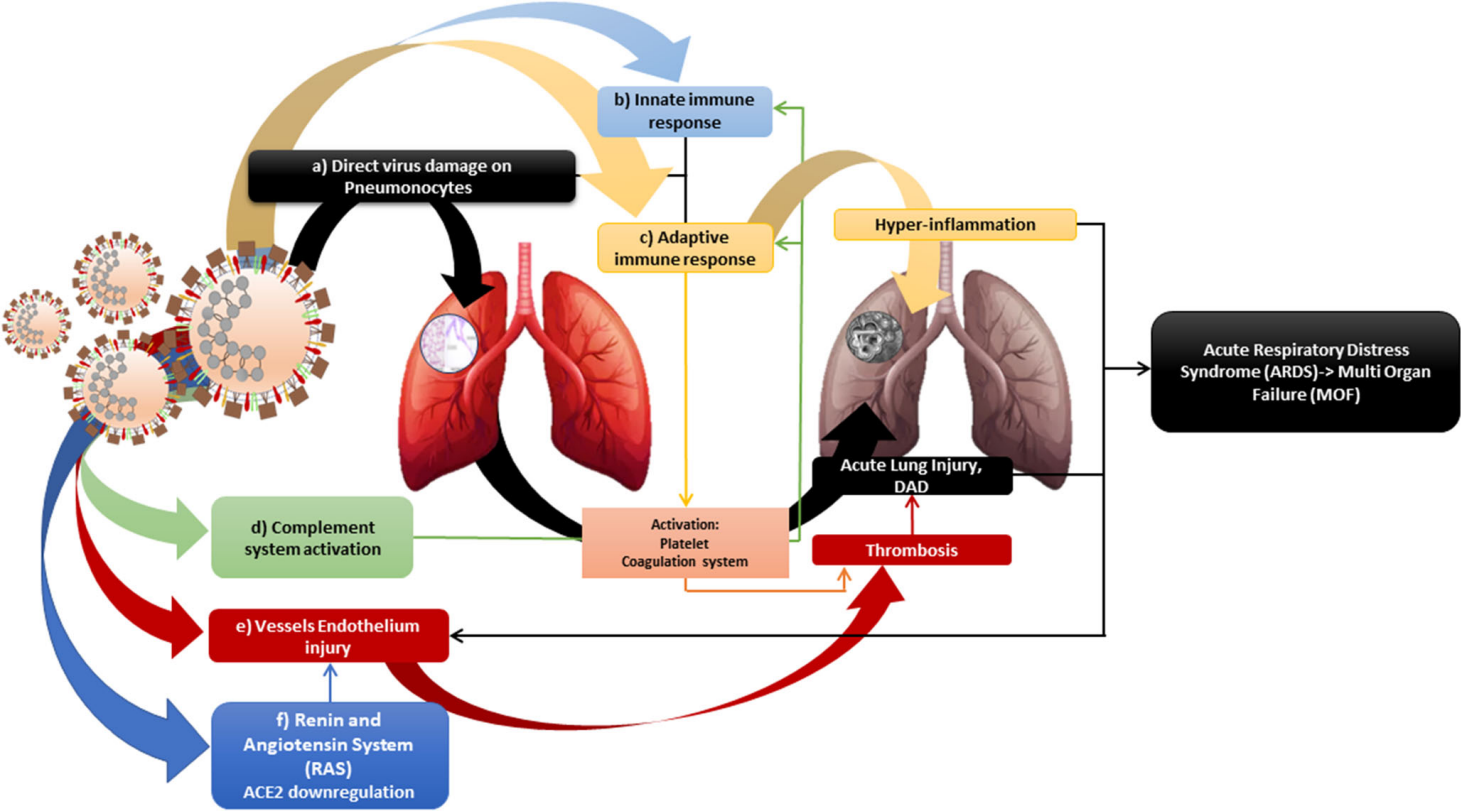
The virus causes direct and indirect effects that lead to severe lung damage. Direct actions: **a** induces damage directly to pneumonocytes I and II with cytopathic consequences through pro-inflammatory status (cytokines/chemokines), **b** recruitment activation of the innate immune response (macrophages/PMN, proinflammatory status (cytokines/chemokines tempest), **c** recruitment activation or the adaptive immune response (antiviral CD8, antiviral B cell, antibodies), **d** complement system (generation of C3a, C5a, membrane complex), **e** endothelial activation and injury leading to endothelial dysfunction and vascular leakage), and **f** RAS: downregulation of ACE2 which increases ACE/AT1 with pro-inflammatory, pro-oxidative effects, vasoconstriction, and pulmonary endothelial damage. Activation of the adaptive immune system causes lung tissue hyper-inflammation, activation of coagulation system (which in turn reactivates complement), activation of platelets (which in turn activates coagulation system), down regulation of inactivity and apoptosis. The activation of the coagulation system and the dysregulation of the ACE2 also lead to endothelial damage thus causing an increase in the possibility of formation of clots and microthrombi/thrombi (such as pulmonary embolism, deep vein thrombosis, large vessel stroke, arterial and venous thromboembolism); increase the risk of lung damage with edema, diffused alveolar destruction (DAD), severe hypoxemia; and can evolve into acute respiratory distress syndrome (ARDS) and afterwards multi organ failure (MOF)

Human pathogenic coronaviruses (SARS-CoV and SARS-CoV-2) bind to their target cells through ACE2, which is expressed by the epithelial cells of the lung, intestine, kidney, and blood vessels (SARS-CoV-2 binds ACE2 with higher affinity than SARS-CoV).The ACE2 is a monocarboxypeptidase that acts on many molecules within the renin-angiotensin system and other substrates, such as apelin (molecule that regulates blood sugar and increases insulin sensitivity). Expression of ACE2 has been reported in type 2 pneumocytes. Functionally, there are two forms of ACE2: (a) “full-length” ACE2 contains a transmembrane domain capable of attaching its extracellular domain to the plasma membrane. The extracellular domain is a receptor that binds to protein S from both SARS-CoV and SARS-CoV-2. The new coronavirus is evolutionarily related to Bat-SARS, which similarly uses membrane-bound ACE2 as a receptor and (b) the soluble form of ACE2 lacks membrane anchorage and circulates in small quantities in the blood. Some authors suppose that this soluble form can act as an interceptor of SARS-CoV-2 (but also for the other coronaviruses) which by acting can prevent the link between S and ACE2 at the cell surface as shown by some in vitro studies. Furthermore, ACE2 combined with a certain portion of Fc immunoglobulin was neutralizing for SARS-CoV-2 in vitro [40, 42, 50]. Thus, the angiotensin system is involved in viral infection. This activity is linked to the blood coagulation cascade. By blocking the ACE2 receptor, severe vasoconstriction occurs, which can lead to the accumulation of fluids in the lungs. There are clotting factors outside the blood vessels (e.g., von Willebrand factor acts on factor VIII) that, when the damage starts to occur as it also happens with the surrounding vessels, enters the blood and this acts on the clotting factors which can cause phenomena of thrombosis. These form in the lungs and cause damage, acute respiratory distress syndrome, stroke, heart disease, and others. ACE2 is one of the main enzymes in the renin-angiotensin system (RAS) which regulates blood pressure, fluids, electrolyte balance, and systemic vascular resistance [42, 50]. In the lungs, activation of local RAS can influence the pathogenesis of lung damage through multiple mechanisms, such as increased vascular permeability and alterations in alveolar epithelial cells. Activation of pulmonary RAS involves renin, the initial enzyme of the RAS activation cascade; renin cleaves angiotensinogen generating angiotensin I (Ang I, a decapeptide hormone, inactive). ACE converts Ang I to angiotensin II (Ang II, an octapeptide hormone), which is very active, which exerts vasoactive effects by binding to its receptors, type I (AT1) and type II (AT2). There is multiple evidence on the importance of AT2 and ACE2 in the regulation of inflammation, both through activation of the AT2 receptor via angiotensin 2, and through activation of the MAS receptor, via Ang 1–7 produced by ACE2, with a reduction of inflammatory interleukins such as interleukin- 6, Il-5, TNF-alpha, and NF-kB. In addition, some studies show that lung ACE2 deficiency correlates with a worse prognosis since ACE2 and AT2 have a protective action in the lung; ultimately, ACE2 is not only the receptor for the virus but also the protector of lung damage [31, 41, 51]. Some evidence shows that SARS-CoV-2 is able to deregulate the balance of the protective system of the lung formed by the balance between ACE/AngII/AT1 and ACE2/AT2/Ang 1–7 and MAS receptor. Is it a possible explanation that the ACE2 receptor in the severity of the infection induced by SARS-CoV2? Several studies have shown that the host’s cells defend themselves from attack by decreasing the presence of ACE2. The expression of ACE2 is substantially increased in patients with type 1 diabetes or type 2 diabetes, arterial hypertension. Consequently, increased ACE2 expression would facilitate COVID-19 infection. Indeed, diabetes and treatment of hypertension with ACE2 stimulatory drugs are hypothesized to increase the risk of developing severe and fatal pneumonia. Finally, the scholars analyzed the presence of ACE2 also to try to clarify the animal origin of the SARS-CoV-2 coronavirus, initially connected to the bat but later also attributed to the pangolin. The analysis carried out showed a greater similarity between the ACE2 protein of human cells and those of pangolins: a result that supports the hypothesis that the small mammal could have been the original host of the SARS-CoV-2 virus or an intermediate host between bat and man [41, 48, 54] (Fig. 4). In bat-SARS-like CoV, the S1 end has a low similarity degree with the equivalent of SARS-CoV, especially in RBD, while the similarity is high at the level of the S2 end and they are unable to use the ACE2 of humans. These differences imply that bat-SARS-like CoVs and SARS-CoVs recognize different molecules on the surface of the host cell as receptor but exploit the same entry mechanism [55, 56]. The theory of the acquisition by genetic recombination of the binding capacity with human ACE2 by bat-SARS-like CoVs is favored by the high genetic variability characterizing the ACE2 of bats, compared to that of other animals currently recognized as sensitive to SARS-CoV. The high diversity of cell receptors in bats strongly suggests the possibility that there is a species of bat not yet characterized that could act as a reservoir for SARS-CoV-19 or for one of its ancestors, as was hypothesized for SARS-CoV [57].

### d) The immunity over time

The virus can be detected by PCR in whole blood and in stool samples (real-time reverse transcriptase-polymerase chain reaction (RT-PCR)), and the serological blood test looks for antibodies created by the immune system [58, 59]. Some aspects regarding the progression of SARS-CoV-2 still remain uncertain. One of these is the role of cross-immunity with other *Coronaviruses* (at least the 4 coronaviruses that cause flu in humans) and following the SARS-CoV epidemic and studies led to the belief that cross-immunity between the common cold virus and SARS-CoV-2 is very likely directed against the antigens common to all coronaviruses and not the specific antigens of SARS-CoV-2 [59, 60]. According to what has emerged so far, all patients who have contracted SARS-CoV-2 and are cured have produced antibodies, but there are conflicting theories on their duration (from a few months to the end of the year). If at first it was thought that antibody immunity for SARS-Cov-2 could last at least a year (as ascertained for the SARS-CoV and MERS-CoV coronaviruses), there may be a drop in plasma antibody levels 2–3 months later healing in both patients who were asymptomatic and in those with symptoms. If so, even those who have already contracted the virus once could become vulnerable after a short time and if the infected quickly lose antibodies, they could be negative on serological tests. However, immunity conferred by antibodies is not the only possible one. In several studies, the immunity mediated by specific T lymphocytes, neutralizing antibodies, and other molecules produced by B lymphocytes capable of neutralizing the virus was also highlighted. In addition, some studies reported the possible enhancement of the immune response by probiotic administration [48, 59–62]. The presence of antibodies, of any type, is not a guarantee of absolute immunity, and the persistence of the antibodies produced remains uncertain [58, 63–66].

### e) Virus influence on human microbiota

Human colonization by microbes in different numbers and compositions begins immediately after birth in any area of the body that comes into contact with the environment, such as the skin; the oral cavity; the rhinopharynx; the lungs; the intestine; the breast; the urogenital system; and, based on recent studies, the placenta and uterine. The microbiota are an organized community of microorganisms, bacteria, fungi, protozoa, which are found in a network of polysaccharides and are attached to a living or inactive surface. An adult human being consists of about 10^13^ cells colonized by 10^14^ microbes. These cells maintain the immune homeostasis for defense that is the eubiosis condition. In some cases, as in viral infections, arises a dysbiosis that creates an imbalance not only locally but also to other systems [67]. These pathways of immune communication (crosstalk) are the axes of the microbiota such as gut/lung, gut/brain, and gut/skin [67–70]. The gut microbiome can be unbalanced (dysbiosis) in many viral inflammation situations such as this in the current SARS-CoV-2 pandemic. Indeed, changes in the fecal microbiome were in fact observed in some infected patients; the genera *Lactobacillus*, *Bifidobacterium*, *Streptococcus*, *Clostridium*, and *Firmicutes* were over represented and the genera *Coprococcus*, *Parabacteroides*, *Roseburia*, *Faecalibacterium*, and *Bacteroidetes* were less. *Firmicutes* have a potential influence on intestinal ACE2 expression and *Bacteroidetes* have shown an inverse correlation with SARS-CoV-2 fecal load and protective effect against inflammation. *Firmicutes* have a potential influence on intestinal ACE2 expression and *Bacteroidetes* have shown an inverse correlation with the fecal load of SARS-CoV-2 which has a potential protective role, and this may be a predictor responsible for the course and severity of the infection. In fact, recent studies show that gastrointestinal pathology in patients with infections that are due to rhinoviruses, adenoviruses, infectious mononucleosis viruses, and coronaviruses (such as the new COVID-19 pandemic) can be more frequent and lasting than pulmonary ones. This can be linked precisely to the imbalance of the intestine/lung axis, or from bacterial dysbiosis, or to an ischemic damage of the central nervous system, but also from interleukin-6 which can also be facilitated by nasopharyngeal dysbiosis [71–75]. The most common skin manifestations associated with SARS-CoV-2 infection include a maculo-papular or papulo-vesicular rash, urticaria lesions, and livedo reticularis. The most common areas involved are the trunk, hands, and feet, with little itching experienced and no proven correlation, between skin lesions and SARS-CoV-2 severity and may be a homeostasis alteration of the gut/skin axis with regard to this gastrointestinal involvement, and as it happens for various other respiratory tract infections that can complicate these disorders. In fact, in some pathologies, recent studies report a correlation between intestinal and cutaneous dysbiosis, which has also been demonstrated in some studies through the administration of probiotics. Generally, the probiotics fight the spread of pathogens, strengthen normal flora, and contribute to the creation of a strong immune system, creating a healthy environment that encourages healing and recovery in a natural way, and this may be useful as an adjuvant therapy in SARS-CoV-2 infection. At last, to modulate an active immune response we expect to have a safe and effective vaccine and a specific therapy for COVID-19 as soon as possible  [72, 76–81].

## Conclusions

The SARS-CoV-2 pandemic emergency has shown the world that only an international scientific effort can stop a threat of this magnitude; also because in some cases, it has shown the whole world the weakness of local health systems. The research effort of the scientific community continues to understand how the new virus works and thus to have an early diagnosis, better management, and adequate therapy. The virus is now well-researched in a short time but the victims around the world continue to be a lot. It causes no or mild symptoms, such as flu-like and taste and smell disorders, but also serious diseases such as the diffuse alveolar damage (DAD), acute respiratory distress syndrome (ARDS), multi organ failure (MOF), and thromboembolic phenomena that make it necessary to study the virus pathophysiology continuously. However, it seems that the virus is “changing” and probably this will make it less pathogenic because it shows the deletion in non-structural proteins (such as NSP1), the most important determinants of the pathogenicity of SARS-CoV-2. On the other hand, cross-immunity and immunity questioned at a certain time that a patient can develop and maintain over time. However, there are no certainties about the future evolution of the virus and its possible disappearance or, on the contrary, its persistence in the population with an enhancement of its virulence. This is important for the risk of reinfection in recovered patients, documented by various cases around the world, and reinforces the need for research for an effective vaccine. Finally, based on the recognized ability of probiotics to stabilize and enhance the resident microbiota and host immune system, as well as on the basis of positive data emerging from ongoing clinical trials on the potential role of probiotics in therapy for SARS-CoV-2, these could be considered both for prevention and to improve the course of the disease.
